# No associations between blood pressure and brain volumes in a convenience sample of Hispanic/Latino middle-aged and older adults

**DOI:** 10.3389/fnagi.2026.1729134

**Published:** 2026-05-05

**Authors:** Carolina L. Costa, Carlos E. E. Araujo-Menendez, Alyssa Lawrence, Alonzo Mendoza, Wassim Tarraf, Ariana M. Stickel

**Affiliations:** 1 Department of Psychology, San Diego State University, San Diego, CA, United States; 2 SDSU/UC San Diego Joint Doctoral Program in Clinical Psychology, San Diego, CA, United States; 3 School of Public Health, San Diego State University, San Diego, CA, United States; 4 Institute of Gerontology & Department of Healthcare Sciences, Wayne State University, Detroit, MI, United States

**Keywords:** blood pressure, brain, Hispanic/Latino, magnetic resonance imaging, older adults

## Abstract

**Background:**

High blood pressure (BP) is a known risk factor for dementia, but the relationships between BP and gross brain volumes are mixed and are understudied in groups who are at particularly high risk for dementia, such as Hispanic/Latino adults. Therefore, we aimed to investigate the relationships between BP and brain volumes, among Hispanic/Latino older adults.

**Methods:**

Participants included 122 cognitively healthy Hispanic/Latino late middle-aged and older adults ages (mean age = 73.48 years, SD = 7.32) from the National Alzheimer’s Coordinating Center. Data were collected from 2005 to 2024 from 20 Alzheimer’s Disease Research Centers. BP measures included systolic (SBP), diastolic (DBP), and pulse pressure (PP). Brain outcomes included total, hippocampal, gray matter, and white matter hyperintensity volumes. Covariates included age, sex, Hispanic/Latino heritage, hypertension, diabetes, hypercholesterolemia, body mass index, hypotensive status, and smoking status.

**Results:**

No BP measure was significantly associated with brain outcomes (all *p* > 0.05).

**Discussion:**

Overall, we found no relationships between BP measures and brain volumes in this sample of late middle-aged and older Hispanic/Latino adults. Our findings warrant longitudinal studies to better characterize the relationships between BP and other cardiovascular disease risk factors with brain health in this population.

## 1 Introduction

High blood pressure (BP), or hypertension, is the primary risk factor for cardiovascular disease (CVD), the leading cause of death globally (Di Cesare et al., 2025; Fuchs and Whelton, 2020). High systolic and diastolic BP (SBP and DBP, respectively) are also associated with an increased risk of cognitive decline, dementia, and cerebrovascular disease (Albanese et al., 2013; Chobanian et al., 2003; Iadecola, 2013; Iadecola et al., 2016; Lennon et al., 2019; Nation et al., 2016). Additionally, mid-life high pulse pressure (PP) is associated with declines in executive functioning (Nation et al., 2016). PP reflects the combined interaction between cardiac stroke volume and aortic compliance (Dart and Kingwell, 2001), and high PP is thought to be indicative of increased arterial stiffness and reduced aortic compliance, which leads to greater load on the cardiovascular system (Safar et al., 2003; Kim, 2025). More broadly, the impact of high BP on the brain is thought to primarily involve disruptions in cerebral blood flow due to deregulation in perfusion pressure (Claassen et al., 2021). High BP can lead to ischemia and cerebral hypoxia by modifying cerebral blood vessels and limiting appropriate nutrients and oxygen reaching the brain (Iadecola and Davisson, 2008).

Although most older adults experience some degree of cerebral atrophy as part of the normal aging process (Fjell et al., 2009), high SBP and DBP are associated with smaller total and regional brain atrophy (e.g., neuronal degeneration) (Beauchet et al., 2013). Additionally, a systematic review with meta-analysis by Alateeq et al. (2021) found that the associations between BP and brain volumes are not limited to individuals who meet clinical diagnosis of hypertension, and among cognitively healthy adults, the relationships between BP and brain volumes are particularly strong for hippocampal and white matter hyperintensity (WMH) volumes (Alateeq et al., 2021). However, the links between BP and brain volumes are inconsistent (Jochemsen et al., 2013; Vlek et al., 2009; Won et al., 2024). For example, greater SBP was associated with higher rates of hippocampal atrophy among individuals with mild cognitive impairment (MCI) and Alzheimer’s disease (Fiford et al., 2020). In contrast, Korf and colleagues found that isolated high SBP and DBP were not associated with hippocampal volume reduction in a sample including cognitively normal and participants with MCI and dementia (Korf et al., 2004).

Contradictory results may also be related to whether the potential confounding effects of other CVD risk factors were accounted for in the associations between BP and brain volume outcomes. Existing literature points to evidence that high BP is often an independent risk factor for cognitive decline and brain atrophy, even in the presence of comorbid CVD risk factors. In Cox and colleagues, high BP was associated with smaller brain volumes even after adjusting for other risks such as obesity, dyslipidemia, or type 2 diabetes mellitus (Cox et al., 2019). Additionally, a systematic review of fifty studies determined that higher BP can influence cognitive outcomes irrespective of other CVD risk comorbidities, such as diabetes (Forte et al., 2020). Furthermore, when compared to lipid levels, obesity, and fasting glucose, high BP is the strongest predictor of cognitive decline (Levin et al., 2014).

Although Hispanic/Latino individuals often face adversities such as discrimination, lower income, and stress related to immigration and acculturation, they sometimes have better health outcomes when compared to their non-Hispanic White counterparts, a phenomenon commonly known as the “Hispanic Paradox” (Markides and Coreil, 1986). However, CVD and brain health is a growing concern for this population. Hispanic/Latino adults are disproportionately affected by dementia and are projected to have the highest increase of Alzheimer’s disease and related dementias (ADRD) in the coming years (Matthews et al., 2019). Data from the 2017–2020 National Health and Nutrition Examination Survey (NHANES) revealed that 56.8% of Hispanic/Latino adults have poorly controlled high BP (National Center for Health Statistics, 2023). Additionally, an estimated 42.7% of Hispanic/Latina females and 52.3% of Hispanic/Latino males over the age of 20 years are affected by CVD (Chobufo et al., 2020; Virani et al., 2021). Lack of appropriate healthcare access may contribute to these disparities. Hispanic/Latino adults have lower rates of medical insurance coverage when compared to non-Hispanic/Latino -Black, -Asian, and -White adults in the US (Petruzzi et al., 2022).

Although the associations between individual BP measures and brain volumes have been examined in the broader population (Fiford et al., 2020; Power et al., 2016; Alateeq et al., 2021), to date, the relationships between SBP, DBP, and PP and specific brain volumes remain underexplored in the Hispanic/Latino population. Considering the increasingly high rates of ADRD within this population (Matthews et al., 2019), the current study aimed to investigate the relationships between BP and brain volumes among Hispanic/Latino late middle-aged and older adults. We hypothesized that higher BP would be associated with smaller brain and larger WMH volumes.

## 2 Materials and methods

### 2.1 Data source

This study utilized data from the National Alzheimer’s Coordinating Center (NACC) Uniform Data Set (UDS) which compiles standardized clinical, neuropsychological, and demographic information collected at approximately annual intervals from participants evaluated at National Institute on Aging–funded Alzheimer’s Disease Research Centers (ADRCs) across the United States. Clinical evaluations are performed by trained personnel using harmonized protocols, and informed consent is obtained at each ADRC (Beekly et al., 2007; Weintraub et al., 2009). In accordance with ADRC protocol, clinical diagnosis of cognitive status was established either by a single physician or an expert-consensus panel (Liampas et al., 2024; Winblad et al., 2004; Petersen et al., 1999; McKhann et al., 1984). Participants were categorized into one category: cognitively normal, cognitive impairment without MCI, cognitive impairment with MCI, or dementia. In this study, only participants who were cognitively normal at the time of their magnetic resonance imaging (MRI) scan were included. BP measurements, as well as medical information were collected during these interviews. The analytic data were drawn from visits collected between 2005 and 2024, with a data freeze in June 2024.

A subset of participants also contributed structural MRI data via NACC’s Standardized Centralized Alzheimer’s & Related Dementias Neuroimaging (SCAN) initiative, which curates structural MRI submissions from ADRCs, links them to UDS records, and distributes centrally processed volumetric measures with quality control metadata (National Alzheimer’s Coordinating Center, 2015a, 2015b). This approach enables pooling across multiple centers with heterogeneous imaging protocols.

### 2.2 Participants and analytic sample

Participants were eligible for inclusion if they self-identified as Hispanic/Latino, were aged 55 years or older, cognitively healthy at the clinical visit used for analysis, and had at least one structural MRI scan that could be temporally matched to a clinical evaluation (see below for details on the matching procedure). Individuals with missing essential covariate data, invalid brain volume measures, or missing intracranial volume (ICV) were excluded.

To align MRI and clinical data, MRI sessions were matched to clinical visits by minimizing the absolute difference in visit dates. For participants with multiple MRI and clinical visits, all possible date pairings were generated. For each participant, the pair with the fewest days between visits was selected first, and this process was repeated iteratively until all MRI and clinical visits were matched. When multiple candidate matches remained, preference was given to pairs with smaller date gaps, cognitively healthy status, and more complete covariate data. Pairs with gaps greater than 12 months were excluded, and only the best match per participant was retained for analysis. This procedure yielded a cross-sectional dataset linking MRI, BP, demographic, and health data. The final analytic sample included 122 Hispanic/Latino adults for the primary sample and up to 209 adults for a sensitivity analysis from 20 ADRCs.

### 2.3 MRI measures and processing

Structural MRI scans were collected across ADRCs using institution-specific protocols; sequence types varied and often included T1-weighted, FLAIR, T2-weighted, diffusion, or other sequences. Imaging parameters, field strengths, and scanner models differed across centers, reflecting the heterogeneity inherent in multisite studies (National Alzheimer’s Coordinating Center, 2015b, 2015a). Submissions to SCAN undergo rigorous quality-control checks for protocol compliance, defacing, and artifact rejection before being accepted for volumetric analysis.

Volumetric analyses were performed centrally by the Imaging of Dementia & Aging (IDeA) Lab at UC Davis using established segmentation pipelines adapted for multisite data. The pipeline produces estimates of brain volumes, including those used in the present study—ICV, total brain, total gray matter, hippocampal (summed across hemispheres), and WMH volumes. Brain volumes were derived from T1-weighted images using atlas-based segmentation and expectation–maximization algorithms; WMH volumes were computed from FLAIR or T2-FLAIR sequences using lesion segmentation algorithms that distinguish hyperintense regions characteristic of small vessel disease (Younes et al., 2023). Because WMH distributions were right-skewed, WMH volumes were log-transformed (natural log) before inclusion in regression models. All brain outcomes of interest were residualized for ICV. That is, using linear regression per brain measure, we generated standardized (z-score) residuals after including ICV as a predictor variable. These residualized brain volumes were then used in the statistical analyses presented below.

### 2.4 Exposure variables

The primary exposures of interest were the clinically assessed SBP and DBP at the MRI-matched visit and PP (calculated as the difference between systolic and diastolic values). During each clinical visit, BP was measured by trained research personnel as part of the standardized NACC Uniform Data Set protocol. Measurements were typically obtained in a seated position after a brief rest period using a manual or automated sphygmomanometer, following the procedures in place at each ADRC. SBP and DBP readings were recorded during the same visit as other clinical assessments and entered into the UDS database (Beekly et al., 2007). These variables were treated as continuous predictors.

### 2.5 Covariates

Demographic and health covariates were selected based on prior literature on vascular and neurodegenerative risk. These included age at clinic visit (at BP reading), sex (male/female), Hispanic/Latino heritage (Mexican/Other), diabetes (present/absent), hypercholesterolemia (present/absent), hypertension status (present/absent), hypotension status (i.e., SBP ≤ 90 or DBP ≤ 40), body mass index (continuous), and smoking history (ever smoked/never smoked). In accordance with ADRC protocol, clinical diagnosis of cognitive status was established either by a single physician or an expert-consensus panel (Liampas et al., 2024; Winblad et al., 2004; Petersen et al., 1999; McKhann et al., 1984).

### 2.6 Derived health variables

Derived health variables were constructed to harmonize risk factor definitions across participants and ADRCs. Hypertension was operationalized using a combination of diagnostic history variables and medication-use indicators (i.e., clinician-reported hypertension, self-reported history, and antihypertensive medication use). Diabetes status was derived from clinician diagnosis variables and glucose-lowering medication indicators. Hypercholesterolemia was similarly obtained using lipid disorder diagnosis and lipid-lowering medication variables. Body mass index was computed using measured weight and baseline or carried-forward height when the body mass index field was missing (body mass index = (Weight (lbs) × 703) ÷ [Height (in)]^2^). A binary smoking history variable was derived using multiple NACC UDS smoking indicators, including recent smoking, lifetime cigarette use, years smoked, and average packs per day. Participants were classified as having ever smoked if any smoking indicator was greater than zero.

### 2.7 Statistical analysis

All analyses were conducted using R version 4.5.1 (R Core Team, 2025). Data management and statistical modeling were performed with several key packages, including tidyverse for data wrangling (Wickham et al., 2019), data.table for efficient MRI–clinical visit matching (Dowle and Srinivasan, 2024), broom for tidying and organizing model estimates (Robinson et al., 2023), and tableone for generating descriptive statistics (Yoshida and Bartel, 2022). Additional packages such as dplyr and ggplot2, both part of the tidyverse ecosystem, were used for data manipulation and visualization (Wickham et al., 2019). Statistical significance was evaluated at a two-sided alpha = 0.05. Linear regressions were run to test the associations between each BP measure (in units of 10 mmHg) with each brain volume outcome in separate analyses. Two nested covariate models were specified. The first model (M1) included age, sex, and Hispanic/Latino heritage. The second model (M2) further included derived indicators of hypertension, diabetes, hypercholesterolemia, body mass index, hypotension status, and smoking history.

### 2.8 Exploratory analyses

We ran exploratory analyses to test for sex differences in the associations between BP measures with brain volumes. To achieve this, sex was included as a moderator in interaction terms with each BP measure in fully adjusted models.

## 3 Results

### 3.1 Descriptives

Table 1 presents the baseline demographic and clinical characteristics of the analytic sample (*N* = 122). The overall sample was 73.48 years old on average and largely comprised of females (*n* = 84; 69%) and those of Mexican heritage (*n* = 86; 71%). The majority (71%) met criteria for hypertension, 30% met criteria for diabetes, 65% met criteria for hypercholesterolemia, and 16% were considered hypotensive. Average body mass index was 29.57, and 40% of the sample met criteria for having a history of smoking. Average SBP, DBP, and PP measures were 139.70, 73.19, and 66.51, respectively.

**Table 1:** Sample descriptives.

Baseline characteristics	Overall
Total, *n*	122
Age, mean (SD)	73.48 (7.32)
Sex (female), *n* (%)	84 (68.9)
Hispanic heritage, *n* (%)	
Mexican	86 (70.5)
Other	36 (29.5)
Hypertension, *n* (%)	
Yes	87 (71.3)
No	35 (28.7)
Diabetes, *n* (%)	
Yes	37 (30.3)
No	85 (69.7)
Hypercholesterolemia, *n* (%)	
Yes	79 (64.8)
No	43 (35.2)
Body mass index, mean (SD)	29.57 (4.85)
Hypotension, *n* (%)	
Yes	19 (15.6)
No	103 (84.4)
Smoking status, *n* (%)	
Yes	49 (40.2)
No	73 (59.8)
Blood pressure measurements, mean (SD)	
Systolic blood pressure	139.70 (19.72)
Diastolic blood pressure	73.19 (10.89)
Pulse pressure	66.51 (20.23)

SD, standard deviation.

### 3.2 Main associations between blood pressure and brain volumes

In primary analyses, no BP measures were significantly associated with any brain outcomes (see Table 2), though higher PP was marginally associated with larger hippocampal volumes (*β* = 0.061, SE = 0.035, *p* = 0.079) and larger gray matter volumes (*β* = 0.70, SE = 0.038, *p* = 0.068) in demographics controlled models but not in fully adjusted models.

**Table 2:** Associations between blood pressure and brain volumes in the cognitively healthy sample.

Brain volume variables	Models			
	M1		M2	
	*β*	SE	*β*	SE
Total brain				
SBP	0.001	0.035	−0.009	0.039
DBP	−0.032	0.062	−0.076	0.092
PP	0.011	0.035	0.004	0.039
Hippocampus				
SBP	0.054	0.035	0.061	0.039
DBP	−0.024	0.062	−0.010	0.094
PP	0.061^†^	0.035^†^	0.064	0.039
Gray matter				
SBP	0.056	0.038	0.060	0.042
DBP	−0.044	0.068	0.022	0.102
PP	0.070^†^	0.038^†^	0.058	0.043
White matter hyperintensities				
SBP	0.070	0.043	0.066	0.049
DBP	0.026	0.077	−0.075	0.119
PP	0.062	0.043	0.081	0.050

^†^*p* < 0.10.

M1: Adjusted for age, sex, Hispanic/Latino heritage. M2: Further adjusted for hypertension, diabetes, hypercholesterolemia, body mass index, hypotension, and smoking status.

*β*, beta estimate; DBP, diastolic blood pressure; M#, model; PP, pulse pressure; SBP, systolic blood pressure; SE, standard error.

*β* represents the change in z-standardized total brain volume associated with a 10-mmHg change in blood pressure.

### 3.3 Exploratory analyses

Exploratory analyses results are detailed in the Supplemental materials.

## 4 Discussion

This study leveraged data from the National Alzheimer’s Coordinating Center (NACC) to examine the associations between BP and brain volumes, among a diverse group of 122 Hispanic/Latino older adults. Contrary to expectation, BP measures were largely unassociated with brain volumes across the sexes. The findings from the current study underscore the need for further investigation into individual differences in the relationships between BP and brain aging among Hispanic/Latino adults.

In contrast to previous findings (Jochemsen et al., 2013; Vlek et al., 2009; Won et al., 2024), we did not find evidence to support relationships between our three BP measures (SBP, DBP, and PP) and brain volumes in our sample. Though a recent meta-analysis (Alateeq et al., 2021) suggested that hippocampal volumes may be particularly sensitive to atrophy in the presence of high BP, including among cognitively healthy individuals, we did not find evidence of this. Previous studies that have also reported null associations between BP measures and hippocampal volumes have included samples with a range of cognitive status (Korf et al., 2004) and alternative BP measures (e.g., mean arterial pressure; Allan et al., 2015). One thing to consider when interpreting our null results is that our convenience sample may be susceptible to survivor and health biases. Participants who were most vulnerable to the harmful effects of high BP may be less likely to participate in this research, especially the MRI portion, due to health burdens or may not have survived to be included in the cohort. As a result, individuals who survived to older age may represent a healthier and more resilient subgroup of Hispanic/Latino older adults. For example, surviving participants may have protective biomarkers or vascular characteristics that reduce the adverse structural brain consequences typically associated with high BP.

The absence of significant associations between BP measures and WMH volumes, a marker of small vessel disease, in the current sample was particularly striking. Recent work that examined the associations between BP and WMHs among females in the Health and Aging Brain Study Health Disparities cohort found that the association between hypertension (broadly defined by self-report, BP measurements, and/or BP medication use) and WMHs was weaker for Hispanic/Latino relative to non-Hispanic/Latino White females (Hayes et al., 2025). Despite this, the same study found that higher PP was significantly associated with larger WMH volumes solely among Hispanic/Latino females but not non-Hispanic/Latino White or Black participants (Hayes et al., 2025). In a sample of Hispanic/Latino adults, we previously found that higher global CVD risk (measured using the Framingham Risk Score) was associated with larger WMH burden, independent of sex (Stickel et al., 2024). Instead, sex appears to play a bigger role in the age-related differences in WMH burden for cognitively healthy Hispanic/Latino adults: females were more susceptible to larger WMH volumes with older age (Stickel et al., 2023). With regard to other brain outcomes, our previous work suggested that medium and high CVD risk was associated with smaller brain volumes, particularly for males (Stickel et al., 2024) whereas a measure of cardiovascular health (Life’s Simple 7 s) was not differentially associated with brain outcomes by sex (Trifan et al., 2025). It is important to note, however, that these models examined aggregate cardiovascular health indicators, which include BP as one of several components, whereas the analyses in the present study focused on the independent effects of BP. Including a composite CVD risk score can obscure the distinct contributions of individual components like BP due to shared variance and potential multicollinearity. Regardless, these findings, as a whole, demonstrate a need to further investigate sex-specific biological mechanisms that may influence the connections between CVD risk factors and brain aging. Indeed, the exploratory findings in the present study suggest potential sex differences in the relationships between SBP and brain volumes (though this was specific to total brain volumes, not WMHs), and these findings need to be replicated in larger samples. Biological mechanisms related to reproductive health, such as changes in estrogen levels during menopause, pregnancy-associated vascular remodeling, and differences in endothelial function, may contribute to greater susceptibility to WMH accumulation and microvascular injury among females (Barnett et al., 1999; Christou et al., 2005; DuPont et al., 2019; Ji et al., 2020; Lakatta and Levy, 2003; Scuteri et al., 2014). Fluctuations in reproductive hormones influence vascular tone, blood–brain barrier integrity, and inflammatory responses, which over time could exacerbate cerebrovascular damage and accelerate white matter aging in females.

A notable strength in our study included the consideration of each BP measurement (SBP, DBP, and PP) separately. Though this did not make a difference in the primary analyses, we did detect differences in the relations between the various BP measures and brain outcomes in exploratory analyses. Similarly, in Ye et al. (2024), high SBP, but not high DBP and PP, was associated with changes in brain structure. These findings raise important questions regarding the differential effects of BP measures on brain health. Due to its higher force, high SBP may have more identifiable effects on brain volumes when compared to high DBP and PP. An additional strength of the present study is that we accounted for Hispanic/Latino heritage, allowing us to comment on our findings broadly across heritage groups. However, given that our sample was primarily of Mexican heritage, we were unable to examine differences by heritage. Different Hispanic/Latino heritage groups can experience distinct risk factors for CVD and social determinants of health that may impact the associations between BP and brain volumes (Elias et al., 2023; Garcia et al., 2021).

Our study had some additional limitations. First, the cross-sectional design limits the ability to draw conclusions about the temporal and causal nature of the observed associations. Future analyses using longitudinal BP data from NACC could help clarify the directionality of these associations and determine if they persist over time. Second, the NACC data are collected throughout multiple centers, possibly contributing to more variability in measurement. However, in earlier versions of the analysis, we controlled for scanner and the results remained (data not shown). Third, our sample size was relatively small, particularly for males, limiting the interpretation and generalizability of our exploratory results. A larger, more balanced sample may yield more robust and generalizable findings, allowing for a better understanding of the observed exploratory associations and thorough correction for multiple comparisons. Fourth, largely due to our limited sample size and lack of available data, we were unable to account for key sociodemographic factors (e.g., socioeconomic status, environmental context) that may influence the relationships between BP and brain volumes in Hispanic/Latino older adults. These factors may shape cardiovascular disease risk and brain health through pathways, such as access to and quality of healthcare, chronic stress exposure, social networks, language barriers, among others (Teshale et al., 2024; Powell-Wiley et al., 2022; Silva-Rudberg et al., 2024). Last, the current study was a convenience sample, in turn, the data may be prone to sample bias (survivor bias) and not reflect the true population, further limiting the generalizability of our results.

## 5 Conclusion

In this sample of 122 cognitively healthy Hispanic/Latino middle-aged and older adults, we found that across sexes, BP measures were not associated with brain volumes. Larger studies are needed to better understand the relationships between cardiovascular health and brain health in this population.

## Data Availability

Publicly available datasets were analyzed in this study. This data can be found at: https://naccdata.org, National Alzheimer’s Coordinating Center (NACC) Imaging Database—Accession No. U01 AG016976; Standardized Centralized Alzheimer’s and Related Dementias Neuroimaging (SCAN MRI) Database—Accession No. U24 AG072122.
