# Supplementary material for: No associations between blood pressure and brain volumes in a convenience sample of Hispanic/Latino middle-aged and older adults
Source: Front Aging Neurosci. 2026 May 5;18:1729134. doi: 10.3389/fnagi.2026.1729134 (PMC13183853; doi:10.3389/fnagi.2026.1729134)
Supplement: Supplementary file 1 [file Table_1.DOCX]

**Supplemental Text:**

***Exploratory analysis results:***

The interaction between sex and SBP on total brain volumes was significant in both Model 1 (β = 0.237, SE = 0.083, p < 0.01) and Model 2 (β = 0.254, SE = 0.088, p < 0.01). However, post-hoc testing revealed that SBP was not significantly associated with total brain volumes in either sex. There were also observed sex differences in PP with total brain volume in both Model 1 (β = 0.186, SE = 0.089, p < 0.05) and Model 2 (β = 0.192, SE = 0.092, p < 0.05), reflecting a similar pattern as seen with SBP for males and females in Model 1. Specifically, the association of PP with total brain volume was trending in the fully adjusted model for males (β = 0.159, SE = 0.083, p = 0.059) and was non-significant for females (β = −0.033, SE = 0.042, p = 0.440). Thus, these findings were not robust, despite these slopes significantly differing by sex (p < 0.05).

When Benjamini-Hochberg FDR corrections were applied to correct for multiple comparisons, the interactions between sex and BP measures on total brain volumes in fully-adjusted models were trending for SBP (corrected p = 0.056) and no longer significant for PP (p = 0.230).

DBP was not associated with any brain outcomes. Finally, no significant associations were observed between any BP indices and WMH volumes, and no sex differences were detected in these associations. See Supplemental Table 1.

| Supplemental Table 1. Sex interactions in the associations between blood pressure and brain volumes in the cognitively healthy sample | | | | | |
| --- | --- | --- | --- | --- | --- |
|  | | | | | |
|  | Models | | | | |
|  |  | | | | |
| Brain Volume Variables | M1 | | | M2 | |
|  | β | SE | | β | SE |
| Total Brain |  |  | |  |  |
| SBP x Sex | 0.237** | 0.083** | | 0.254** | 0.088** |
| DBP x Sex | 0.196 | 0.136 | | 0.195 | 0.149 |
| PP x Sex | 0.186* | 0.089* | | 0.192* | 0.092* |
|  |  |  | |  |  |
|  |  |  | |  |  |
| Hippocampus |  |  | |  |  |
| SBP x Sex | 0.034 | 0.085 | | 0.070 | 0.091 |
| DBP x Sex | 0.033 | 0.137 | | 0.014 | 0.152 |
| PP x Sex | 0.047 | 0.089 | | 0.077 | 0.093 |
|  |  |  | |  |  |
|  |  |  | |  |  |
| Gray Matter |  |  | |  |  |
| SBP x Sex | 0.036 | 0.093 | | 0.059 | 0.100 |
| DBP x Sex | 0.223 | 0.149 | | 0.229 | 0.165 |
| PP x Sex | -0.029 | 0.098 | | -0.017 | 0.102 |
|  |  |  | |  |  |
|  |  |  | |  |  |
| White Matter Hyperintensities |  |  | |  |  |
| SBP x Sex | -0.152 | 0.105 | | -0.173 | 0.115 |
| DBP x Sex | -0.137 | 0.173 | | -0.194 | 0.194 |
| PP x Sex | -0.094 | 0.112 | | -0.095 | 0.118 |
|  |  | |  | | |
| *Note*. * and ** indicate *p* < 0.05 and *p* < 0.01, respectively. † = p < 0.10  M1: Adjusted for age, sex, Hispanic/Latino heritage. M2: Further adjusted for hypertension, diabetes, hypercholesterolemia, body mass index, hypotension, and smoking status.  Abbreviations: β, beta estimate; DBP, diastolic blood pressure; M#, model; PP, pulse pressure; SBP, systolic blood pressure; SE, standard error.  β  represents the change in z-standardized total brain volume associated with a 10-mmHg change in blood pressure. | | | | | |
